# Unbiased analysis of spatial learning strategies in a modified Barnes maze using convolutional neural networks

**DOI:** 10.1038/s41598-024-66855-8

**Published:** 2024-07-10

**Authors:** Tomer Illouz, Lyn Alice Becker Ascher, Ravit Madar, Eitan Okun

**Affiliations:** 1 The Leslie and Susan Gonda Multidisciplinary Brain Research Center, Bar-Ilan University, 5290002 Ramat Gan, Israel; 2 The Mina and Everard Goodman Faculty of Life Sciences, Bar-Ilan University, 5290002 Ramat Gan, Israel; 3 The Paul Feder Laboratory on Alzheimer’s Disease Research, Bar-Ilan University, 5290002 Ramat Gan, Israel; 4 The Leslie and Susan Gonda Multidisciplinary Brain Research Center, The Mina and Everard Goodman Faculty of Life Sciences, Bar Ilan University, Building 901, Room 312, 5290002 Ramat Gan, Israel

**Keywords:** Spatial memory, Cognitive ageing

## Abstract

Assessment of spatial learning abilities is central to behavioral neuroscience and a useful tool for animal model validation and drug development. However, biases introduced by the apparatus, environment, or experimentalist represent a critical challenge to the test validity. We have recently developed the Modified Barnes Maze (MBM) task, a spatial learning paradigm that overcomes inherent behavioral biases of animals in the classical Barnes maze. The specific combination of spatial strategies employed by mice is often considered representative of the level of cognitive resources used. Herein, we have developed a convolutional neural network-based classifier of exploration strategies in the MBM that can effectively provide researchers with enhanced insights into cognitive traits in mice. Following validation, we compared the learning performance of female and male C57BL/6J mice, as well as that of Ts65Dn mice, a model of Down syndrome, and 5xFAD mice, a model of Alzheimer’s disease. Male mice exhibited more effective navigation abilities than female mice, reflected in higher utilization of effective spatial search strategies. Compared to wildtype controls, Ts65Dn mice exhibited delayed usage of spatial strategies despite similar success rates in completing this spatial task. 5xFAD mice showed increased usage of non-spatial strategies such as Circling that corresponded to higher latency to reach the target and lower success rate. These data exemplify the need for deeper strategy classification tools in dissecting complex cognitive traits. In sum, we provide a machine-learning-based strategy classifier that extends our understanding of mice’s spatial learning capabilities while enabling a more accurate cognitive assessment.

## Introduction

Spatial learning is an essential cognitive function that enables organisms to navigate and learn about their surroundings^1^. Indeed, the ability to acquire, store, and use spatial information is crucial for survival in many species, including mice and humans. Further, studying spatial learning in mice is vital for understanding mechanisms that underlie neurodegenerative diseases such as Alzheimer's disease (AD) and Down syndrome (DS)-related AD^2,3^, as well as potential treatments^4^. Spatial learning depends significantly on various types of hippocampal and para-hippocampal cells, including place cells, grid cells, head direction cells, and conjunction cells^5–7^. Engagement of place cells in spatial encoding relies on using distal extramaze visuals cues as inputs for triangulation procedure that dictates excitation in their preferred spatial fields^6,8,9^. Therefore, when designed correctly, these spatial learning tasks can be used to measure the integrity and function of hippocampal-associated networks. To fully assess the spatial cognitive abilities of mice in these pathologies, it is important to consider the complexities of their behavior. Mice utilize different spatial strategies to navigate their environment^10^. It is crucial to understand the specific spatial strategies employed by mice under different physiological and pathological conditions, as different strategies can indicate cognitive abilities or deficits^11,12^. Various highly effective tasks that assess spatial learning and memory in rodents have been described, including the Morris water maze (MWM)^13^, radial arm maze (RAM)^14^, radial arm water maze (RAWM)^15^, and the Barnes maze (BM)^16^. For two of the most widely-used tasks, the MWM and the BM, we have previously developed online tools for classifying behavioral spatial strategies in the MWM^12^ and the BM^11^ using supervised machine-learning algorithms. Additionally, tools for characterization and classification of general and specific rodent exploratory behaviors were previously introduced by several groups, utilizing different computational approaches^17–21^. These classifiers are superior to human manual classification, which tends to be biased, labor intensive, and depends on the degree of expertise of the human classifier^11^. However, since each spatial learning tasks exhibits inherent specific disadvantages, we previously developed a modified variant of the classical BM (MBM)^22^. Specifically, the MBM combines the continuous nature of the MWM while avoiding water-related stress^22^. The MBM enables high flexibility in task difficulty, along with overcoming inherent biases towards non-spatial strategies that are typical of the traditional BM task. In the present study, we describe the development of an unsupervised machine learning algorithm used to classify behavioral strategies in the MBM. We demonstrate the efficacy of this algorithm in classifying spatial strategies in four experimental settings: changing task difficulty, comparing male and female mice, and comparing two neurodegenerative mouse models, namely, AD and DS to wildtype (WT) controls. These four experimental settings represent physiological conditions in which subtle differences are expected, as well as pathological conditions in which significant cognitive impairments are observed, showcasing the dynamic range of strategy classification described herein.

## Methods

### Animals

8-weeks-old Female and male C57BL/6J WT mice (n = 10) were purchased from Jackson Laboratories (stock #000664). Ts(17^16^)65Dn (Ts65Dn), a widely used mouse model for DS that encompasses a partial trisomy of Mmu16 and Mmu17, thus containing 92 genes orthologous to Hsa21, including mouse *amyloid precursor protein (APP)* and *dual-specificity tyrosine phosphorylation-regulated kinase (DYRK1A)*^23,24^, and their background strain (B6EiC3Sn.BLiAF1/J) were purchased from the Jackson Laboratories (stocks #005252, #003647). Mice were tested at the age of 8 months (n = 14). 5xFAD mice on a C57BL/6 genetic background, expressing mutant human *APP* and *presenilin-1 (PSEN1)* genes (B6.Cg-Tg; APPSwFILon, PSEN1*M146L*L286V), were purchased from Jackson Laboratories (stock #034848) and were tested at the age of 8 months (n = 9 for 5xFAD and 10 for WT). Animals were housed in a reversed 12:12 h cycle. All tests were conducted during the dark phase. Proper control groups were chosen based on genetic background of the experimental group in accordance with the Jackson Laboratories strain-specific recommendation. Animals that presented signs of pain or severe stress, including immobility, weight loss and reduced social interaction were excluded from the experiments, in accordance with the Bar Ilan University Animal Care and Use Committee.

Animal care and experimental procedures followed Bar Ilan University’s guidelines and were approved by the Bar Ilan University Animal Care and Use Committee. All experiments were done in accordance with the recommendations of the ARRIVE guidelines.

### Modified Barnes maze

The MBM consisted of a circular, 110 cm-high, 122 cm-wide white Perspex table with 40 randomly placed holes, each with a diameter of 5 cm, located at least 7 cm from each other and at least 5 cm from the perimeter. Six holes were fabricated to function as an optional escape chamber. Lighting was measured at the center of the table and maintained at > 900 lx to motivate the animals to search a target hole that leads to a hidden escape chamber. During a 1-day habituation, animals were placed in a cylinder located 10 cm from the edge of the maze, farthest from the target hole location. of the maze. Five seconds later, the cylinder was removed, and the mice were allowed to explore the environment for 2 min. Mice that found the target hole could enter the escape chamber, while mice that did not find it within this period were placed back in the cylinder, now located above the target hole. Visual cues were presented on the walls surrounding the apparatus. In the spatial acquisition phase, mice were given 2 min per trial to find the target hole. Mice that did not find it were confined to the target hole area until they located it. In this task, each animal was given 3 trials with a 30-s inter-trial interval. The maze was cleaned with 40% EtOH between trials. This procedure was repeated daily until no significant improvement in performance was identified. Performance parameters in this task were automatically calculated by the ANY-maze video tracking system (Stoelting Co.). Specifically, we recorded the latency to reach the target (in seconds), distance travelled (in meters), average speed (m/s), path efficiency, calculated as the ratio between the animal’s trajectory the distance between the start and target points, number of entries to non-target holes (count), time in non-target holes (s), reference memory errors, indicated by the number of entries to non-target holes (count), working memory errors, defined as the number of entries to non-target holes minus the number of reference memory errors (count), absolute angles, calculated as the sum of the absolute angle between each movement vector of the animal, number of rotations (count), calculated as an unbroken sequence of turns in the same direction. All experiment were conducted in a blind manner such as the experimenter was agnostic of animal allocation to experimental group.

### Image processing

X, Y coordinates of the animals’ location throughout each trial were extracted from ANY-maze. All further processing was done in MATLAB (Mathworks). Trajectories were plotted as a black line on a white background, and the target location was indicated by a red dot. MBM table boundaries and holes were not plotted. A set of randomly selected images representing ~ 10% of the of total number of samples was used for initial identification of strategies characteristic of the MBM. This data set also served for hyperparameter optimization. The remaining MBM trials were randomly divided into train (80%) and test sets such that the test set contained 20% of the samples from each label. Test set samples were only used once. For train set samples, images were augmented ten times by five 72° rotations and an additional horizontal flip.

### Convolutional neural networks

Convolutional neural networks (CNN) were trained to classify exploration strategies using the MATLAB Deep Learning Toolbox (Mathworks). A hierarchical classification architecture was implemented as described in the results section. The architecture of each CNN consists of an input layer and multiple repetitions of convolution, batch normalization, ReLU, and max pooling layers followed by fully connected, soft-max and classification output layers. At testing, accuracy rate per strategy was calculated as the fraction of correctly classified strategy out of the total number of samples in that class.

### Statistical analysis

The data presented as mean ± SEM were tested for significance in repeated measures (RM) two-way ANOVA or one-way ANOVA using Tukey’s test for multiple comparisons. All error bars presented are SEM calculated as $\frac{{{\text{std}}({\text{x}})}}{{\sqrt {\text{n}} }}$ for all numerical variables, and as $\sqrt {\frac{{{\text{p}}(1 - {\text{p}})}}{{\text{n}}}}$ for all binomial variables. Animal tracking errors and outliers were removed using the Robust Regression and Outlier Removal (ROUT) method with coefficient Q = 1%^25^. Significant results were marked according to conventional critical P values: *P < 0.05, **P < 0.01, ***P < 0.001, ****P < 0.0001.

## Results

### Identification of distinct spatial learning strategies in the MBM

**Figure 1:**
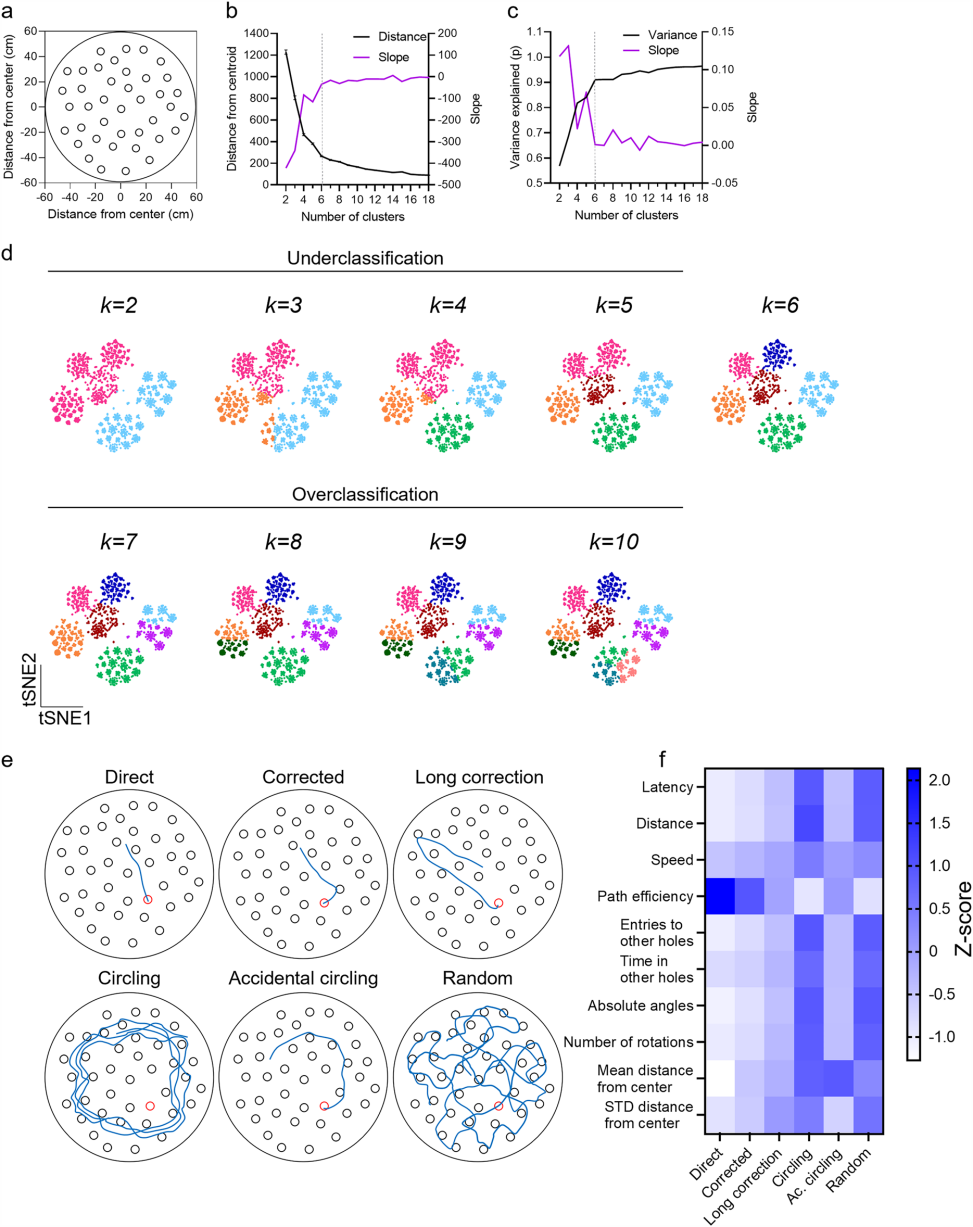
Identification of distinct spatial learning strategies in the MBM. The number of learning strategies used by mice in the MBM was estimated using unbiased techniques. 1508 MBM trials were subjected to *k-me*ans clustering (*k* = 2:18). (**A**) Scheme of the MBM table. The elbow method was utilized by evaluating the elbow point of (**B**) the distance to the centroid and (**C**) the percentage of variance explained by clustering. (**D**) tSNE projection of 1508 MBM trials clustered increasing *k.* (**E**) Pseudo-trajectories typical of the six identified learning strategies*.* (**F**) Averaged Z-score of the variables used to differentiate learning strategies. *STD* Standard deviation.

The MBM combines the spatial continuity of the MWM with the advantages of a dry test environment, a key virtue of the BM (Fig. 1a)^22^. Since the MWM and the BM share some exploration strategies^11,12,22^, we hypothesized that the strategies that characterize mice’s performance in the MBM would reflect the combined nature of this apparatus. To investigate this, we tested whether distinct exploration patterns can be identified using unsupervised learning methods. First, principal component analysis (PCA) was conducted on ten commonly used variables that were extracted from a data set of 1508 trials of C57BL/6J tested in the MBM (Fig. S1a). This set of variables includes latency, distance, average speed path efficiency, number of entries and time spent in non-target holes, absolute angles, number of rotations, mean and standard deviation of the animals' distance from the center of the maze. Variables were calculated as described in the methods section. PCA revealed that linear combinations of variables could reflect different exploration patterns (Fig. S1b,c). For example, principal component (PC) 1, which explains 61.4% (Fig. S1b) of the variance in our dataset, negatively correlated with mice’s path efficiency, calculated as the ratio between the animal’s trajectory the distance between the start and target points, and positively correlated with all other variables (Fig. S1c). This finding indicates that a significant fraction of the variance in this dataset originates from differences between long-insufficient and short-efficient performances (Fig. S1d). PC3 positively correlated with the average distance of the mice from the maze center and negatively correlated with the standard deviation of that distance, indicating that PC3 is a good indicator for distinguishing between focal/random and circular searches. Next, we assessed the number of potential exploration strategies using the elbow method: The average distance of each datapoint from its nearest *k-means* cluster centroid and the variance explained by clustering were calculated on the same ten-dimensional dataset, with the increasing number of clusters (*k*). Both the distance from the nearest centroid (Fig. 1b) and the variance explained (Fig. 1c) were reduced when the data was clustered using $k=2 to 6$ clusters, while only minor changes in these metrics were measured using $k> 6$. Two-dimensional tSNE projection following *k-means* clustering confirmed that the data is indeed under-classified when using $k< 6$, as major clusters were identified as belonging to the same class, and over-classified when using $k> 6$, as major clusters were divided into different classes (Figs. 1d, S1e). Collectively, these data suggests that six clusters are an accurate number of exploration strategies found in the MBM.

**Figure 2:**
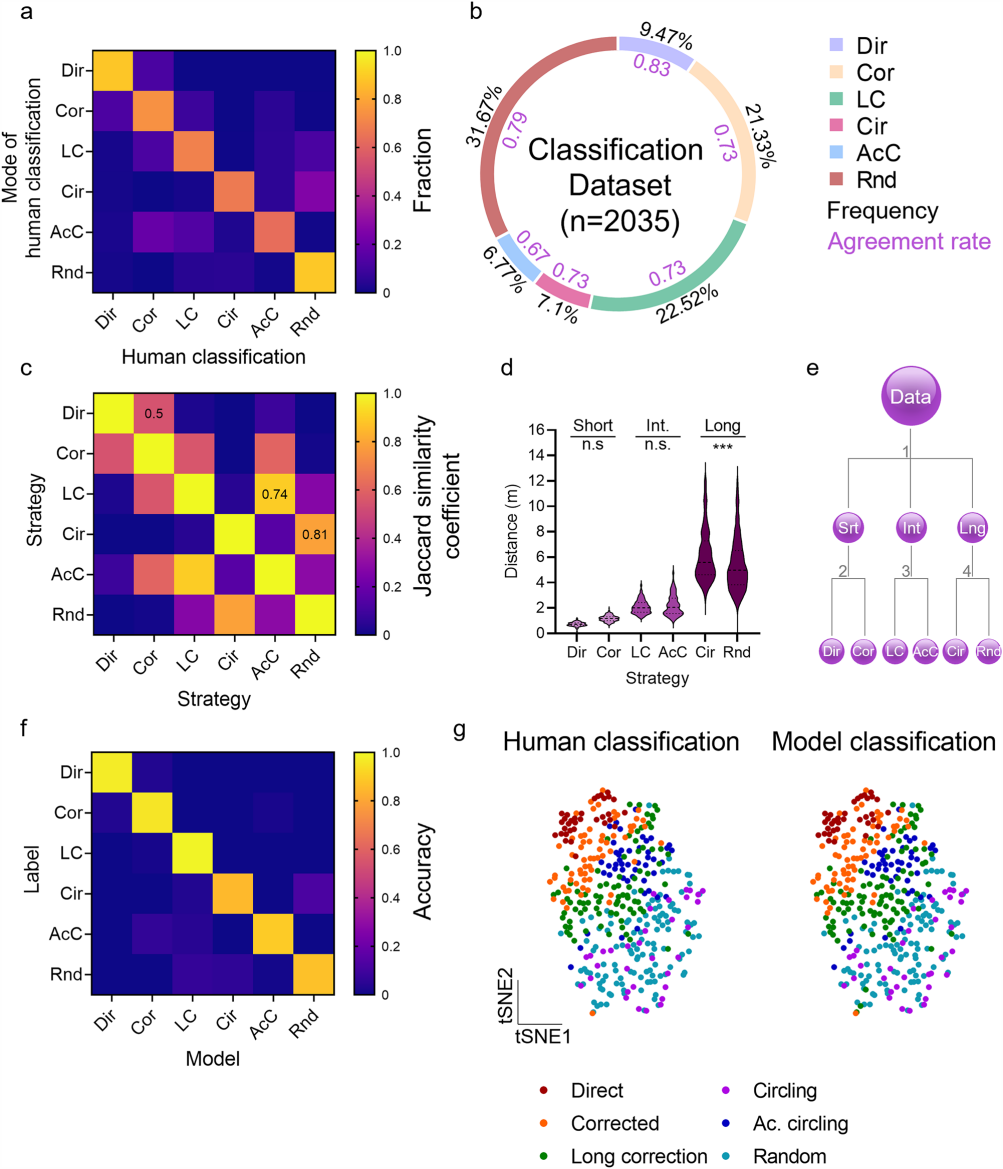
Classification of exploration strategies in the MBM using convolutional neural networks. 2035 MWM trials were subjected to manual labeling by seven individuals to train and test the performance of a neural-network classifier. (**A**) Confusion matrix of all manual labeling versus their mode reveals that *Direct* and *Random* are the most coherently identified strategies. (**B**) Prevalence of different learning strategies obtained from human labeling, averaged between-judge agreement levels, are indicated in purple. (**C**) Pairwise Jaccard similarity coefficients were used to identify pan-categories. *Direct* and *Corrected*, *Long correction* and *accidental circling*, and *Circling* and *Random* were paired into three pan-categories based on a high Jaccard similarity coefficient (indicated within the confusion matrix). These pan categories were defined as (**D**) *short, intermediate,* and *long* trajectories and were then divided into individual strategies on the (**E)** classification dendrogram. (**F**) Accuracy level of the hierarchical neural-network classifier. (**G)** tSNE projection of the test set; human (left) and neural-network model (right) show high similarity. One-way ANOVA, ***P < 0.001, *Dir* Direct, *Cor* Corrected, *LC* Long correction, *AcC* Accidental circling, *Rnd* Random.

Next, trajectory plots of 211 randomly sampled MBM trials were presented to seven human classifiers experienced in conducting spatial learning tasks for human labeling. Individuals were allowed to classify each trial to one of the previously defined sets of strategies characteristic of the MWM^12^ (Fig. S2a) and the BM^22^ (Fig. S2b). The final label of each trial was determined as the mode of human classifications (Fig. S3a). Interestingly, the classification of some strategies was more consistent between human classifiers (e.g., *Direct*, 0.78 agreement level, Fig. S3a), while others were less decisive (e.g., *Long correction,* 0.68 agreement level, Fig. S3a). Since no trial was classified as a *Serial search* by any human classifiers, this strategy was removed from downstream analysis. Next, to meet the optimal number of exploration strategies (Fig. 1b–d), the six most prevalent exploration strategies were selected for full dataset labeling (Figs. 1e,f, S3b). These strategies consist of the *Direct,* in which animals use a relatively straight path from the start point to the target. This strategy reflects optimal acquisition of the environment; *Corrected,* which represent trials that include one turn; *Long Correction,* in which the animal chose exploring the opposite direction from the target, then re-angulated went directly to the target; *Circling*, a non-spatial strategy to enhance the chance of finding the target by exploring the environment in a circular manner; *Accidental circling*, represent circular searches in which the target was found before the first full circle was completed; and *Random search* (Fig. 2e)^11,12^*.* As hypothesized, some MBM exploration strategies overlapped with MWM strategies (*Circling, Accidental circling*). *Long correction* was shared between the BM and the MBM, and three MBM strategies overlapped with both MWM and BM (*Direct, Corrected, Random*, Fig. S3c).

Using the same methodology, a set of 2035 MBM trials, obtained from testing C57BL/6J mice using a non-central target location, was classified by 7 individuals to one of the six predetermined exploration strategies. The final label per each sample was determined using the winner-takes-all approach. Agreement rate between human classifiers, defined as the frequency of the mode label per trial, ranged between 62% for *Accidental circling* and 88% for *Random search* (Fig. 2a,b), indicating that some strategies are more easily identified than others. Interestingly, *Random search* was the most prevalent strategy in this dataset (31.67%), while *Accidental circling* was the least prevalent strategy (6.77%, Fig. 2b). A two-dimensional tSNE projection of this dataset reveals that strategies are ordered between two poles: *Direct* at the upper-left corner and *Random search* at the lower-right corner (Fig. S3d). This suggest that exploration strategies in the MWM can be seen as a spectrum, ranging from the highly spatial strategies (*Direct, Corrected, Long correction*) to the non-spatial strategies (*Circling, Accidental circling* and *Random*, Fig. 2e). In agreement with this idea, the lower-right pole populates trials obtained at early stages of animal training, in which *Random search* is more prevalent, and the upper-left pole populates trials obtained at later stages of animal training, in which *Direct* and *Corrected* searches are more common (Fig. S3e).

### Classification of exploration strategies in the MBM using convolutional neural networks

To obtain a generalized classifier independent of feature selection, variable calculation, and apparatus size, we chose to use CNNs with images of animals’ trajectories as inputs. CNNs are a deep learning neural network commonly used in computer vision tasks. CNNs are designed to automatically detect and learn spatial hierarchies of features from input images or data. They consist of multiple layers of filters that convolve with the input image to extract relevant features such as edges, textures, and shapes. With their ability to automatically learn and extract features from images, CNNs are particularly effective in object recognition and classification tasks^19,26^.

In multi-category classifications, it is often preferable to use hierarchical rather than flat architectures, in which highly similar categories are first treated as pan-categories (https://ieeexplore.ieee.org/document/7410671). In the next level in the classification dendrogram, such pan-categories can be treated separately to deal with highly similar categories. Indeed, we observed strong similarities between some of the MBM strategies. Using pairwise Jaccard similarity indices (JSI), we found strong similarities between *Circling* and *Random* (JSI = 0.8, Fig. 2c), *Long correction* and *Accidental circling* (JSI = 0.74, Fig. 2c), and *Direct* and *Corrected* strategies (JSI = 0.5, Fig. 2c). These pairs were pooled into pan-categories that corresponded to trajectory length (in meters): *Direct* and *Corrected* were pooled into a pan-category of short trajectories, (0.73 ± 0.01, 1.17 ± 0.01 m, respectively, P = 0.07, Fig. 2d), *Long correction* and *Accidental circling* were pooled into an intermediate-length pan-category (2.1 ± 0.02, 2.2 ± 0.06 m, respectively, P = 0.97, Fig. 2d), and *Circling* and *Random* were pooled into a long trajectory pan-category (6.19 ± 0.2, 5.51 ± 0.1, P = 0.0002, Fig. 2d). Path length significantly differed between pan categories (Fig. S4a). Based on these similarities, we devised a two-level hierarchical architecture in which classification into pan-groups is followed by classification into individual categories (Fig. 2e).

Next, a dataset of 2035 MBM trials was randomly divided into train and test sets (80% and 20%, respectively). For the training set, data augmentation was performed by 72° image rotations and a horizontal flip, yielding 10 images per each original sample. Next, we trained a neural network for each classification junctions in the dendrogram (Fig. 2e). Classification accuracy, measured by comparing the model results with the human-labeled test set, reached 91.86% by averaging the percentages of true positive classifications (Fig. 2f–g, S4b,c). Balanced accuracy, calculated as the mean of recalls, reached 91.99%. No significant difference was found in classification-explained variance between human and machine classification (Fig. S4d). As a reference, we trained a Random Forest classifier on the same datasets and obtained a classification accuracy of 76.38% (Fig. S4e), indicating that the CNN was superior to a random forest classifier when tested against human observers.

### Target hole location affects task difficulty and alters the usage of spatial strategies

**Figure 3:**
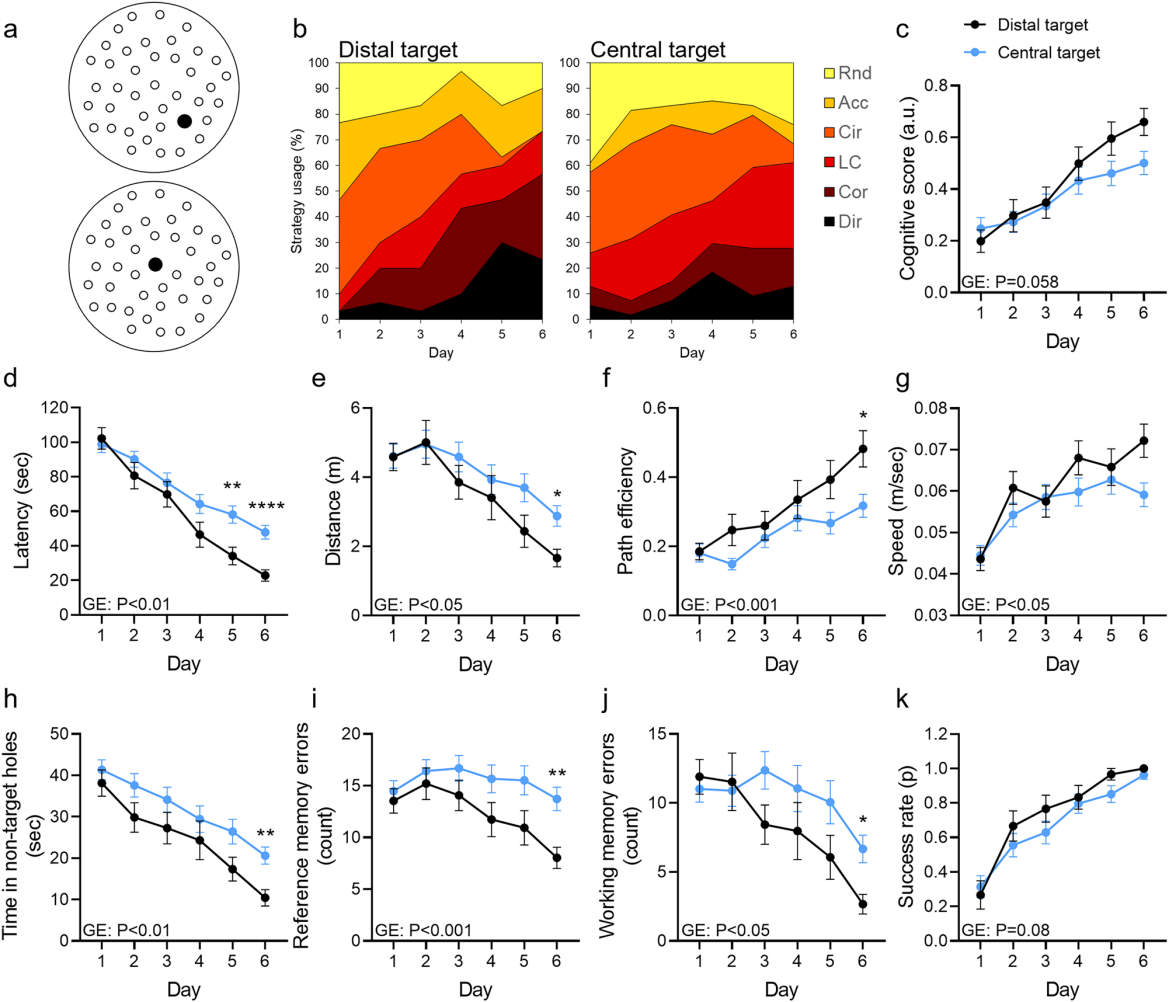
Target hole location affects task difficulty and alters the usage of spatial strategies. (**A**) Male C57BL/6J mice (aged 8 weeks, n = 10) were trained in the MBM using a central and a more distal target location. (**B**) Strategy usage throughout the training was assessed using a neural-network classifier and (**C**) was quantified by the cognitive score scaling. Between-group differences were measured for (**D**) latency to reach the target, (**E**) exploration distance, **(F)** path efficiency, (**G**) walking speed, (**H**) time in non-target holes, (**I**) reference and (**J)** working memory errors, and (**K**) success rate. Repeated-measures two-way ANOVA, *P < 0.05, **P < 0.01, ****P < 0.0001. *GE* Group effect.

We previously observed that the difficulty of the MBM task could be manipulated by using central targets for a more difficult task and distal/peripheral targets for an easier task, allowing the experimenter to adjust task difficulty according to experimental needs^22^. To validate this finding using the strategy classifier, we trained eight-week-old WT male mice (n = 10 per group) in the MBM using a central and an off-center (distal) target (Fig. 3a). As expected, mice that were trained to find the distal target used the *Direct* and *Corrected* strategies at a higher prevalence on the sixth day of training compared with mice trained to find the central, more difficult target (56.67%, 27.77%, respectively, P < 0.0001, Fisher’s exact test, Fig. 3b). Interestingly, mice that were trained to find the central target used mostly *long correction* by the last day of training, suggesting that conversion to the higher *Correction* strategy was beyond the cognitive capacity of mice under this task difficulty level. To further quantify these differences, we established a scoring system for spatial cognition that localizes the animals’ performance on a scale relative to the averaged *direct* performance (ADP) in the MBM. To define this non-arbitrary scale, we calculated the Euclidean distance between each trial in the training set to the ADP. The distance of each strategy was defined as the mean of distances to the ADP per strategy. These results were rescaled to fit the 0–1 range and yielded the following cognitive scores: *Circling* = *0*, *Random* = *0.1*, *Accidental circling* = *0.32*, *Long correction* = *0.61*, *Corrected* = *0.77*, and* Direct* = *1.* The cognitive score did not significantly differ between groups (P = 0.058, Fig. 3c) due to similar scores at the early stages of training. These results reflect a slower learning curve of mice when using the central target. Consistently, latency to target entry, exploration distance, and path efficiency were higher in mice trained to find the central target than in mice trained to find the distal target (P < 0.01, P < 0.05, P < 0.001, respectively, Fig. 3d–f), whereas exploration speed was mildly lower for mice trained to find the central target (P < 0.05, Fig. 3g). Additionally, mice trained to find the central target exhibited elevated time in non-target holes (P < 0.01, Fig. 3h) and an increase in reference and working memory errors (P < 0.001, P < 0.05, Fig. 3i–j). Accordingly, the trajectories of mice trained to find the central location covered a higher percentage of the surface of the MBM table (P < 0.05, Fig. S5a–d). However, success rate did not differ between groups (P = 0.08, Fig. 3k). In sum, we provided evidence that manipulation of target location in the MBM can be used to control task difficulty while affecting the combination of the spatial strategies utilized by mice. This feature of the MBM enables the experimenter to adjust task difficulty to comply with the experiment’s requirements.

### Male C57BL/6J mice exhibit a more effective navigation ability than females in the MBM

**Figure 4:**
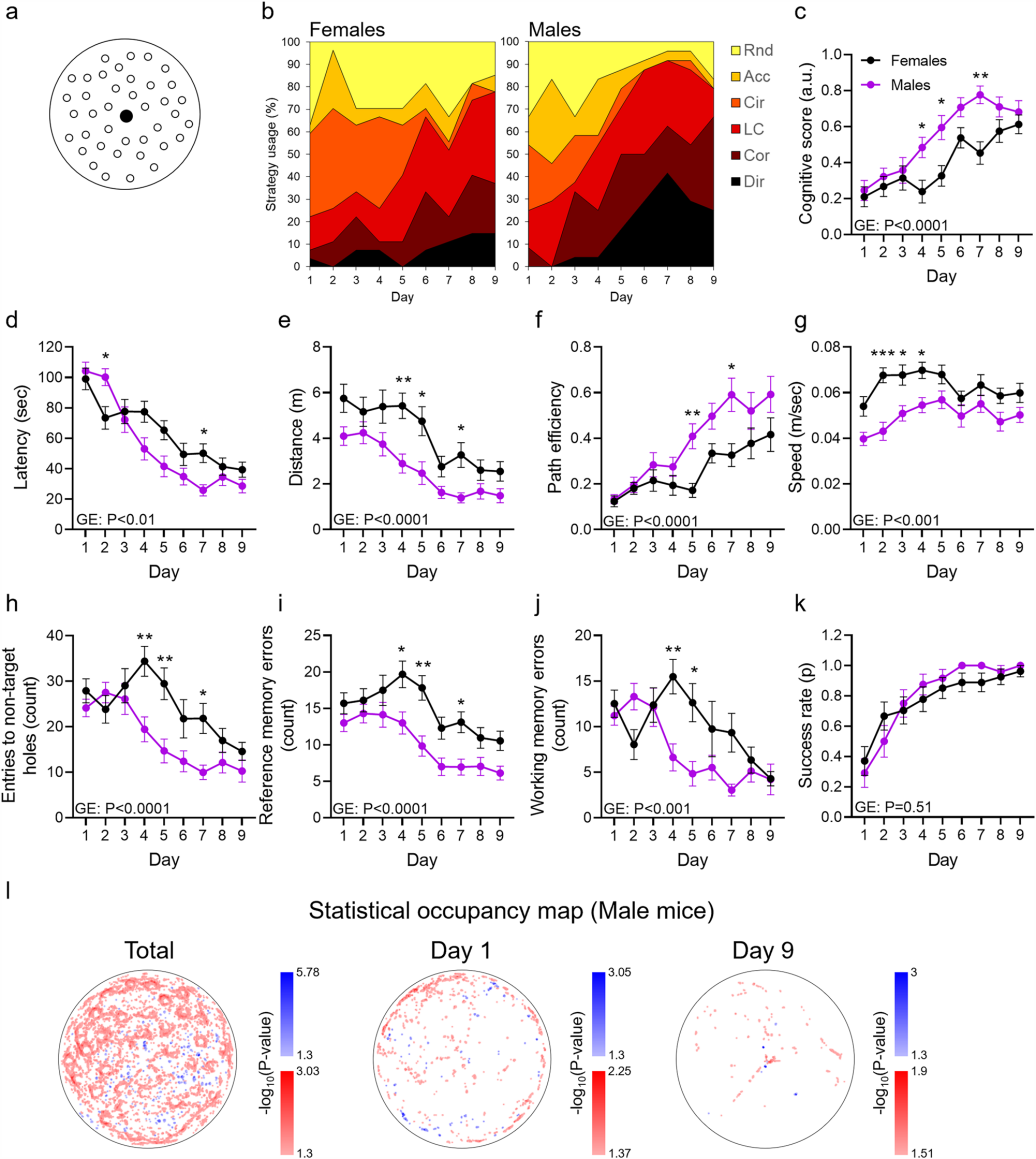
Male C57BL/6J mice exhibit a more effective navigation ability compared with females in the MBM. (**A**) Female and male C57BL/6J mice (aged 8 weeks, n = 10 per group) were trained in the MBM with the hidden escape box placed at the center of the arena. (**B**) Strategy usage throughout the training was assessed using a neural-network classifier and (**C**) was quantified by the cognitive score scaling. Inter-sex differences were observed for (**D**) latency to reach the target, (**E**) exploration distance, (**F**) path efficiency, (**G**) walking speed, (**H**) number of entries to non-target holes, **(I)** reference memory errors, and (**J**) working memory errors but not for (**K**) success rate. (**L**) Statistical occupancy map for all (left), first (middle), and last (right) days of training. Bin-wise change in occupancy of male compared with female mice is indicated in blue (positive fold-change) or red (negative fold-change). P value is coded by the colors’ darkness. Non-significant differences are not shown. Repeated-measures two-way ANOVA, *P < 0.05, **P < 0.01, ***P < 0.001, *GE* Group effect.

With respect to spatial abilities, males outperform females in both murine and in humans, with the underlying mechanisms not entirely clear^27–29^. To assess whether sex-related changes in mice performance in the MBM can be identified using our classifier, female and male C57BL/6J mice (n = 10 per group) were trained for nine days in the MBM. Since no significant deficit in spatial learning abilities was expected in these experimental groups and to enable the identification of subtle differences, we used the most difficult MBM setting in which the target is located at the central hole of the MBM table^22^ (Fig. 4a). On days 1–4 of the training, *Circling* was the most prevalent strategy used by female mice (40.74% at day 4, Fig. 4b), while long correction was the most prevalent strategy used by male mice at this timepoint (29.16% at day 4, Fig. 4b). By the seventh day, 91.67% of male trials were classified as strategies that reflect efficient acquisition of the target location (i.e., *Direct*, *Corrected*, *Long correction*) while these strategies represented 51.85% of the female trials (P < 0.0001, Fisher exact test, Fig. 4b). *Random search* represented 33.33% of the trials at this timepoint among females and only 4.16% among males (P < 0.0001, Fisher exact test, Fig. 4b). These changes were also reflected in higher spatial cognitive scores in males than in females (P < 0.0001, Fig. 4c).

Earlier conversion from non-spatial to highly spatial strategy in males than is females was associated with reduced latency to target entry (P < 0.01, Fig. 4d), reduced exploration distance (P < 0.0001, Fig. 4e), and higher path efficiency (P < 0.0001, Fig. 4f). Intriguingly, females exhibited elevated exploration speed (P < 0.001, Fig. 4g). Exploration accuracy, indicated by the number of entries to non-target holes, or reference memory errors, and working memory errors, defined as the number of entries to non-target holes minus the number of reference memory errors, was also reduced in females compared with males (P < 0.0001, P < 0.0001, P < 0.001, respectively, Fig. 4h–j). Consistently, the area covered by exploration trajectories was higher in females than in males (P < 0.0001, Fig. S6a–d), indicating more scattered searches by females. Success rate, however, did not differ between male and female mice (P = 0.51, Fig. 4k), implying that the usage of less efficient strategies is compensated by increased speed in female mice (Fig. 4g).

To further compare exploration patterns between male and female mice, we segmented the MBM environment into 3 × 3 mm bins and calculated the fold-change (and P values) in occupancy of male versus female mice in a bin-wise manner. These data may be represented as statistical occupancy maps and volcano plots (Figs. 4l, S6e). Stronger spatial learning performance in males was reflected by a shorter and more focused exploration pattern, while females exhibited a more scattered exploration pattern (Figs. 4l, left panel, S5e left panel). While no significant difference was observed on the first day of training (Figs. 4l, middle panel, S6e middle panel), females continued to explore the periphery of the MBM surface by the last day of training (Figs. 4l, right panel, S6e right panel). Altogether, we identified higher spatial learning accuracy in male compared with female mice tested in the MBM.

### Characterization of spatial learning deficits in the Ts65Dn mouse model of DS in the MBM

DS, caused by a trisomy in human chromosome 21 (Hsa-21), is the most common chromosomal abnormality in humans and the most prevalent genetic cause of intellectual disability^30^. Hsa-21 contains approximately 233 protein-coding genes, 423 non-protein coding genes, and numerous other functional genomic elements^30^. The Amyloid precursor protein (APP) gene, located within Hsa-21, is triplicated in DS, such that APP is over-expressed in affected individuals with DS compared with euploid individuals^31^. This heightened expression results in APP-dependent Alzheimer-like neuropathology in ~ 88% of all individuals with DS by the age of 65^31^. The Ts65Dn mouse model of DS encompasses a partial trisomy of mouse chromosome 16, which includes 92 genes orthologous to Hsa21. As a result, this model recapitulates many of the cognitive, behavioral, structural, and physiological abnormalities of DS^23^.

**Figure 5:**
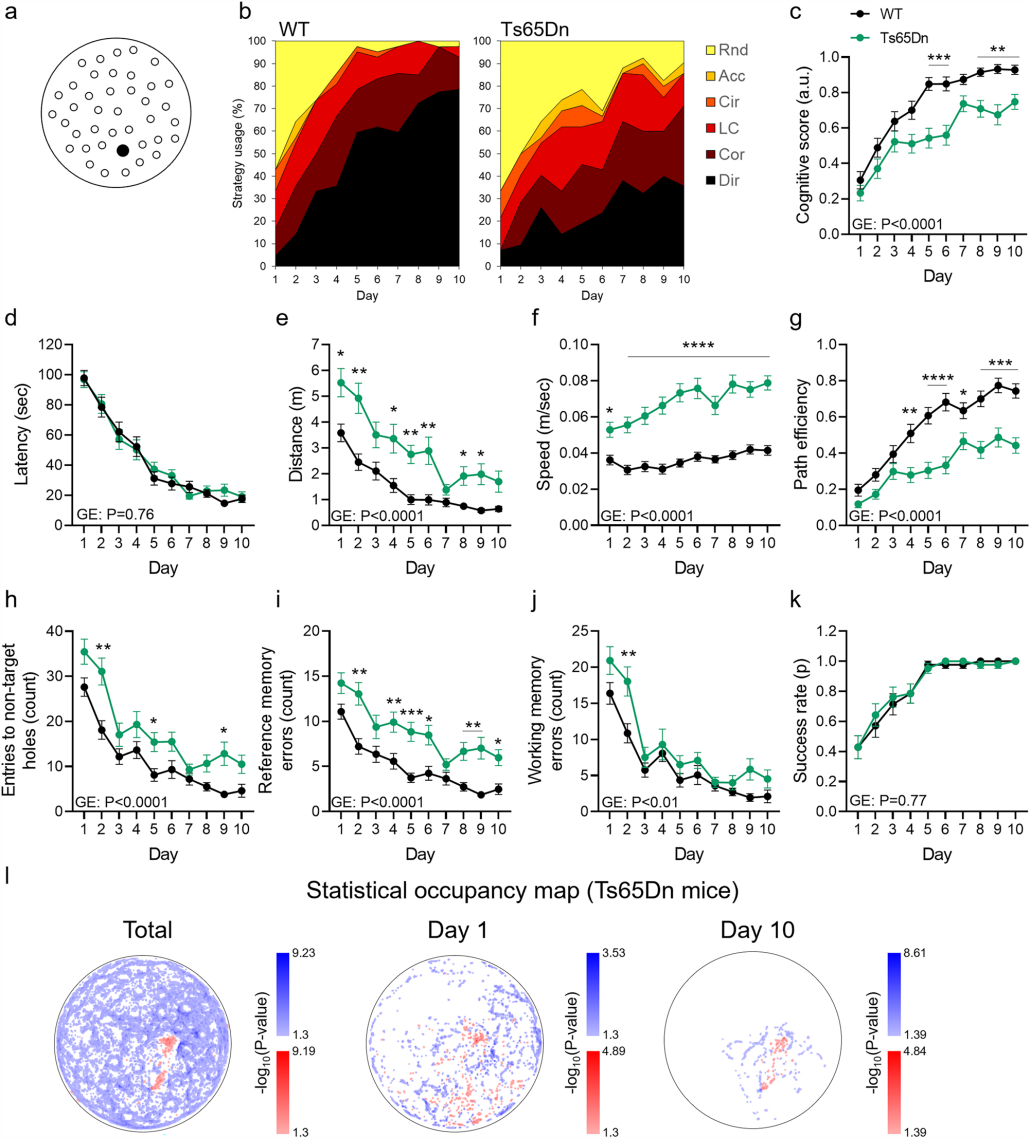
Characterization of spatial learning deficits in the Ts65Dn mouse model of DS in the MBM. (**A**) Male Ts65Dn and WT mice (aged 8 months, n = 14 per group) were trained in the MBM with the hidden escape box placed mid-way between the center and the periphery of the arena. (**B**) Strategy usage throughout the training was assessed using a neural-network classifier and (**C**) was quantified by the cognitive score scaling. Inter-strain differences were measured for (**D**) latency to reach the target, (**E**) exploration distance, (**F**) walking speed, (**G**) path efficiency, (**H**) number of entries to non-target holes, (**I**) reference and (**J**) working memory errors, and (**K**) success rate. (**L**) Statistical occupancy map for all (left), first (middle), and last (right) days of training. Bin-wise change in occupancy of Ts65dn compared with WT mice is indicated in blue (positive fold-change) or red (negative fold-change). P value is coded by the colors’ darkness. Non-significant differences are not shown. Repeated-measures two-way ANOVA, *P < 0.05, **P < 0.01, ***P < 0.001, ****P < 0.0001. *GE* Group effect.

To characterize the cognitive deficits of Ts65Dn mice, we investigated their performance in the MBM using our trained classifier. Eight-month-old male Ts65Dn and their respective genetic background control strain (n = 14 per group) were trained in the MBM for 10 days using a medium difficulty level achieved by placing the escape hole between the periphery and the center of the apparatus (Fig. 5a). Although learning was observed throughout training in both groups, Ts65Dn mice exhibited a profound spatial learning deficit compared to WT controls, reflected in reduced usage of the highly spatial strategies: *Direct*, *Corrected*, and* Long correction* (Fig. 5b). By the fifth day, these strategies represented 95.2% of the WT trials and 61.9% of the Ts65Dn mice trials (P < 0.0001, Fisher’s exact testm, Fig. 5b) By the last day, 9.52% of Ts65Dn trials were classified as *Random*, compared with 2.38% *Random* searches in WT controls (P < 0.05, Fisher’s exact test, Fig. 5b). Accordingly, the cognitive scores of Ts65Dn mice were lower than those of WT mice throughout training (P < 0.0001, Fig. 5c). Importantly, latency to reach the target hole entry did not differ between groups (P = 0.76, Fig. 5d), which corresponded with higher distance (P < 0.0001, Fig. 5e), higher speed (P < 0.0001, Fig. 5f), and lower path efficiency (P < 0.0001, Fig. 5g) in Ts65Dn mice compared with WT controls. These findings provide an example of the need for comprehensive analysis of mice performance in spatial learning tasks beyond comparison of latencies. Ts65Dn mice also exhibited reduced spatial accuracy, indicated by a higher number of entries to non-target holes (P < 0.0001, Fig. 5h), a profound reference memory deficit (P < 0.0001, Fig. 5i), and a milder working memory impairment (P < 0.01, Fig. 5j). Additionally, the exploration trajectories of Ts65Dn mice covered a higher percentage of the MBM table (P < 0.0001, Fig. S7a–d). However, success rate did not differ between groups (P = 0.77, Fig. 5k), indicating compensation of less efficient exploration strategies by higher exploration speed. Using statistical occupancy maps, we found that Ts65Dn mice spent significantly less time in the vicinity of the target location throughout training (Figs. 5l, left panel, S7e, left panel). Accordingly, they spent more time near the periphery of the table on the first day (Figs. 5l, middle panel, S7e, middle panel), and exhibited less target-oriented exploration on the last day of training (Figs. 5l, right panel, S7e, right panel). In sum, our findings indicate a clear spatial learning impairment in the Ts65Dn mouse model of DS due to a deficit in reference memory capacity associated with using less-efficient exploration strategies and increased exploration speed.

### Association of spatial working memory impairment and circular explorations in the 5xFAD mouse model of Alzheimer disease

**Figure 6:**
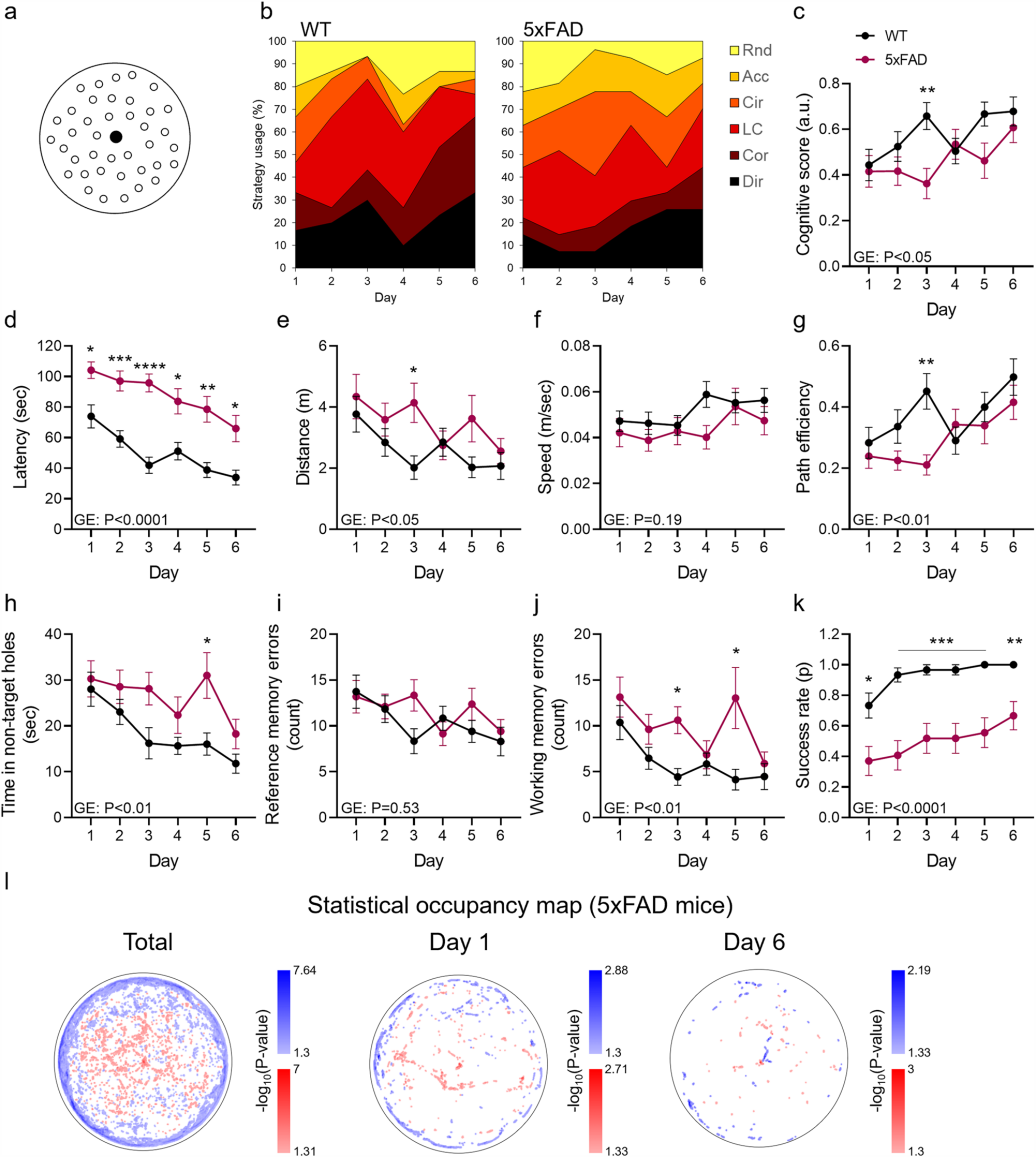
Association of spatial working memory impairment and circular explorations in the 5xFAD mouse model of Alzheimer disease. (**A**) Male 5xFAD and WT mice (aged 8 months, n = 9 per group) were trained in the MBM with the hidden escape box placed at the center of the arena. (**B**) Strategy usage throughout the training was assessed using a neural-network classifier and (**C**) was quantified by the cognitive score scaling. Inter-strain differences were measured for (**D**) latency to reach the target, (**E**) exploration distance, (**G**) walking speed, (**G**) path efficiency, (**H**) time in non-target holes, (**I**) reference and (**J**) working memory errors, and (**K**) success rate. (**L**) Statistical occupancy map for all (left), first (middle) and last (right) days of training. Bin-wise change in occupancy of 5xFAD compared with WT mice is indicated in blue (positive fold-change) or red (negative fold-change). P value is coded by the colors’ darkness. Non-significant differences are not shown. Repeated-measures two-way ANOVA, *P < 0.05, **P < 0.01, ***P < 0.001, ****P < 0.0001. *GE* Group effect.

Spatial learning ability heavily relies on the integrity of hippocampal and para-hippocampal brain regions^32^. The hippocampus is also specifically vulnerable to Alzheimer disease (AD) pathology^33^. Therefore, we investigated the impact of Amyloid-β (Aβ) pathology on the spatial strategy utilization of transgenic AD mice in the MBM. The 5xFAD mouse strain, which models early-onset AD, encompasses five early-onset AD-related mutations: the Swedish (K670N, M671L), London (V717I), and Florida (I716V) mutations in APP and the M146L and L286V mutations in Presenilin 1 (PS1)^34^. As a result, 5xFAD mice exhibit early and profound Aβ pathology in the brain. Eight-month-old 5xFAD (n = 9) and their respective WT control male mice (n = 10) were trained to find the central (most difficult) target of the MBM for 6 days (Fig. 6a). 5xFAD mice exhibited reduced usage of highly spatial strategies (i.e., *Direct*, *Corrected*, and *long correction*) by the third day of training compared with WT controls (40.74%, 83.33%, respectively, P < 0.0001, Fisher’s exact test, Fig. 6b). The most prevalent strategies in 5xFAD mice were *Circling* and *Accidental circling*, represent together 55.55% of strategies used. The performance of WT mice reached an a plateau on day 5, with 80% of WT trials classified as highly spatial. In comparison, 5xFAD mice used these strategies at a prevalence of 44.44% (P < 0.0001, Fisher’s exact test, Fig. 6b). Accordingly, the overall cognitive score of 5xFAD mice was lower compared to WT controls (P < 0.05, Fig. 6c). Unlike the performance of Ts65Dn mice, 5xFAD mice exhibited increased latency to target entry (P < 0.05, Figs. 5d and 6d, respectively), while only mild difference in exploration distance (P < 0.05, Fig. 6e) and no difference in speed was observed (P = 0.19, Fig. 6f). Path efficiency was also reduced in 5xFAD mice compared with controls (P < 0.01, Fig. 6g), but was only associated with early stages of training. Importantly, lower accuracy was observed in 5xFAD mice, indicated by elevated time in non-target holes (P < 0.01, Fig. 6h). Interestingly, reference memory capacity of 5xFAD mice did not differ from WT controls (P = 0.53, Fig. 6i), but working memory capacity was significantly reduced in these mice (P < 0.01, Fig. 6j), which resulted in lower success rate compared with WT mice (P < 0.0001, Fig. 6k). Accordingly, statistical occupancy maps analysis revealed that exploration trajectories of 5xFAD mice covered a higher percentage of the MBM table surface (P < 0.05, Fig. S8a–d), with a clear tendency to explore the periphery of the surface (Fig. 6l, S8e). Overall, we report a working memory impairment in 5xFAD mice trained in the MBM, which is associated with a higher prevalence of the *Circling* and *Accidental circling* strategies.

## Discussion

The MBM is a novel modified variant of the traditional BM task for spatial learning^22^. It combines the advantages of the MWM and the BM while avoiding their disadvantages^22^. As a result, the MBM avoids water stress and its related technical complications, including lengthy operation times characteristic of the MWM. It also avoids non-spatial strategies, such as circling, that are characteristic of the BM. As with more traditional spatial learning tasks such as the MWM and the BM, in which spatial strategy classifiers provide additional layers of information to be extracted^11,12^, we set out to generate an unbiased classifier that effectively classifies cognitive strategies in the MBM, as a tool for the research community.

The algorithm presented herein can effectively analyze MBM data obtained from different transgenic mice with and without cognitive impairments in an unbiased manner while providing a cognitive score scale that assesses memory acquisition.

Traditionally, performance in spatial learning tasks is analyzed according to one-dimensional parameters such as path efficiency, working and reference errors, and latency to reach the target. However, focusing solely on these parameters fails to fully capture the animal’s spatial cognitive capacity. We argue that utilizing a spatial learning paradigm superior to traditionally used paradigms (e.g., the MWM or the BM), combined with an added layer of information on the spatial strategies utilized by the rodents, is advantageous to optimizing experimental efficacy and promoting research output.

In humans and rodents, most sex-difference studies suggest that males outperform females in spatial learning tasks like the MWM and BM^27,35–38^ with no clear understanding of the underlying mechanisms. In rodents, males exhibit lowered escape latency, shorter exploration distance and reduced number of memory and reference errors^27,35,38^. However, male- or female-advantaged experimental environment and protocols may bias such reports. Indeed, sex-related tendency for spatial learning strategy is one proposed mechanism that explains these differences. Males show a tendency to use geometric cues, a place strategy, while females exhibit tendency towards relying on visual landmarks such as a visible platform^39,40^. Intriguingly, these behaviors are highly dependent on sex hormones. Our finding supports the hypothesis that males and females utilize different exploration strategies. However, to test whether these strategies correspond to place and cued strategies, extinction of a familiar target location and acquisition of a new target using landmarks should be tested. Interestingly, our data also suggests that despite clear differences in path length, number of errors and averaged speed, male and female mice do not differ in their success rate to find the MBM target.

In addition, the approach can sensitively identify behavioral nuances in pathologically relevant conditions, such as spatial learning deficits found in DS and AD mouse models. Various transgenic, knock-in and injection mouse models of AD have been extensively used in research as a tool of understanding disease mechanisms, biomarkers and for testing novel therapeutics paths^41,42^. In many of these studies, cognitive ability, and specifically hippocampus-dependent spatial learning and memory abilities serve as major redouts. Spatial learning task such as the MBM and BM has been extensively used to this end. However, correctly modeling and evaluating cognitive deficits in mouse models of AD may be challenging. Sex-differences in AD pathology are considered central in understanding the great heterogeneity seen in patients. These differences in neural anatomy, disease prevalence and progression as well as cognitive manifestation, suggest that different disease promoting mechanism operate in men and women^43,44^. Given that in rodents, males and females may exhibit a tendency towards using a different sets of exploration strategies, these differences should be taken under consideration in testing cognitive deficits in the context of mouse models of AD. Similarly, motor co-morbidities^22^, age differences at testing, the combination of physiological cognitive aging and cognitive decline in AD, as well as different disease mechanism that operate in the different models^41^ call for deeper evaluation of the cognitive phenotype that goes beyond one-dimensional variables as latency or path length.

Here we compared two models of cognitive deficits, the Ts65Dn mouse model of DS and 5xFAD model of AD to their genetic background WT strains. In Ts65Dn mice, our data provide evidence for slower acquisition of the target location, reflected in milder and delayed conversion from ‘lower’ to ‘higher’ navigation strategies as compared to WT mice. Interestingly, no differences in latency, as well as success rate were observed in these mice, while exploration speed was elevated, suggesting that different navigation strategies are used in the by the two groups. It is therefore possible that the reported motor hyperactivity in this strain^45^ interacts with cognitive deficit to yield this distinct behavioral phenotype. It is, however, unclear whether the observed increased exploration speed in Ts65Dn mice acts negatively on spatial encoding functions or serves as a compensation mechanism for the reduced ability to encode the target location. As the ‘high’ spatial strategies emerged in later days of the test, we hypothesize that the inherent motor hyperactivity act as a confounding effect on evaluating cognitive deficits in this mouse model.

In the 5xFAD mouse model of AD we observed increased prevalence of trials that were classified as ‘Circling’ and ‘Accidental circling’, alongside with increased latency and reduced success rate. These data suggest that in contrast to the Ts65Dn model, 5xFAD mice compensate for their reduced ability to encode the environment by using non-spatial, chance-increasing strategies. Therefore, we suggest that conversion from non-spatial to spatial strategies, and specifically the prevalence of using the ‘Circling’ strategy is a relevant readout when evaluating this strain.

The limitation of this study include lack of assessment of the estrous cycle^46^, limited sample size, and inter- but not intra-experimental condition differences, such as the gender of the experimenter, the season, table orientation within the testing room. Additionally, no assessment of anxiety or exploratory behavior was conducted. Due to limited number of trials of the less frequent classes, strategies were not balanced in our data set. Therefore, balanced accuracy was reported alongside the percentage of true positive classification.

In summary, the algorithm presented herein offers the research community an improved, powerful, and precise tool for assessing spatial learning in rodents.

### Supplementary Information

Supplementary Information 1.

Supplementary Information 2.

Supplementary Information 3.

Supplementary Information 4.

Supplementary Information 5.

Supplementary Information 6.

Supplementary Information 7.

Supplementary Information 8.

Supplementary Information 9.

## Data Availability

All data including a user interface will be available upon request. Requests should be addressed to Prof. Eitan Okun, PH.D., (Eitan.okun@biu.ac.il) or Tomer Illouz, Ph.D., tomerillouz@gmail.com).
