# Supplementary material for: Unbiased analysis of spatial learning strategies in a modified Barnes maze using convolutional neural networks
Source: Sci Rep. 2024 Jul 10;14:15944. doi: 10.1038/s41598-024-66855-8 (PMC11237060; doi:10.1038/s41598-024-66855-8)
Supplement: Supplementary file 1 — Supplementary Information 1. [file 41598_2024_66855_MOESM1_ESM.docx]

**Supplementary figure legends**

**Figure S1. Principal component analysis reveals different exploration patterns in the MBM.** 1508 MBM trials were subjected to principal component analysis (PCA) to identify meaningful linear combinations of commonly used metrics. (**A**) Two-dimensional PC projection. (**B**) fraction of variance explained by PCs. (**C**) Pearson’s correlation of each variable with the different PCs indicates different exploration patterns in the MBM. (**D**) Scatter plot of animals’ path efficiency versus latency to reach the target (left) and distance travelled (right) (**E**) Averaged Z-score of the different variables following *k-means* clustering using an increasing number of clusters (*k*). Abbreviations: principal component (PC); standard deviation (STD).

**Figure S2.** **Spatial learning strategies in the MWM and the BM.** Pseudo-trajectory plots representative of previously defined learning strategies in the (**A**) MWM and (**B**) the BM. These strategies were used as optional categories for trial labeling by human classifiers. Abbreviations: Morris water maze (MWM); Barnes maze (BM).

**Figure S3. Defining spatial strategies in the MWM by human classifiers.** 211 randomly selected MWM trials were presented to seven individuals with prior experience in behavioral spatial learning testing. Individuals were given the option to classify each trial to previously defined strategies in the MWM and the BM. The final label was determined using the winner-takes-all approach. (**A**) Confusion matrix of the Mode versus all labels. (**B**) Prevalence of different learning strategies obtained from human labeling, averaged between-judge agreement levels, are indicated in purple. (**C**) The MBM strategies were defined as the six most prevalent strategies in the defining set, which overlap with some strategies previously defined for the MWM and BM. (**D**) tSNE projection of 2035 MBM trials with color coding for the six learning strategies characteristic of the MBM and (**E**) day of acquisition.

**Figure S4. Classification of exploration strategies in the MBM using convolutional neural networks.** Hierarchical neural-network classifier was trained to distinguish (**A**) pan categories, defined as *short, intermediate,* and *long* trajectories. (**B**) Accuracy level of the model versus human labeling at each classification node on the dendrogram. (**C**) human and model tSNE projection following the first (short, intermediate, and long), second (direct and corrected), third (long correction and accidental circling), and fourth (circling and random) classification nodes. The overall classification is presented in the bottom panels. (**D**) Percentage of variance explained by human and model classification do not show a difference. (**E**) Lower classification accuracy was obtained using a Random Forest classifier.

**Figure S5. Target hole location affects task difficulty and alters the usage of spatial strategies.** (**A**) Overlayed trajectory plots and (**B**) occupancy plots at the first (upper panels) and last day (lower panels) of the acquisition phase, with the hidden escape box location indicated (upper and lower right panels, respectively). (**C**) Percentage of the MBM table covered by trajectories and (**D**) fraction of time mice occupied an increasing radius around the target at the first and last days. Repeated-measures two-way ANOVA, Abbreviations: Group effect (GE).

**Figure S6. Male C57BL/6J mice exhibit more effective navigation compared with females in the MBM.** **(A)** Overlayed trajectory plots and (**B**) occupancy plots on the first and last day of the acquisition phase, with the location of the hidden escape box located at the center of the arena (right panel). (**C**) Percentage of the MBM table covered by trajectories and (**D**) fraction of time mice occupied an increasing radius around the target on the first and last days. (**E**) Volcano plots of the bin-wise occupancy of male mice compared with female mice used to generate the statistical occupancy maps; statistically significant bins are marked in color. Repeated-measures two-way ANOVA, *P<0.05, **P<0.01, Abbreviations: Group effect (GE).

**Figure S7. Characterization of spatial learning deficits in the Ts65Dn mouse model of DS in the MBM. (A)** Overlayed trajectory plots and (**B**) occupancy plots on the first and last day of the acquisition phase, with the hidden escape box located midway between the center and the periphery of the apparatus (right panel). (**C**) Percentage of the MBM table covered by trajectories and (**D**) fraction of time mice occupied an increasing radius around the target on the first and last days. (**E**) Volcano plots of the bin-wise occupancy of Ts65Dn compared with C57BL/6J mice used to generate the statistical occupancy maps; statistically significant bins are marked in color. Repeated-measures two-way ANOVA, *P<0.05, **P<0.01, ***P<0.001 Abbreviations: Group effect (GE).

**Figure S8. Association of spatial working memory impairment and circular explorations in the 5xFAD mouse model of Alzheimer disease.** (**A**) Overlayed trajectory plots and (**B**) occupancy plots on the first and last day of the acquisition phase, with the location of the hidden escape box located at the center of the arena (right panel). **(C)** Percentage of the MBM table covered by trajectories and (**D**) fraction of time mice occupied an increasing radius around the target on the first and last days. (**E**) Volcano plots of the bin-wise occupancy of 5xFAD compared with C57BL/6J mice used to generate the statistical occupancy maps; statistically significant bins are marked in color. Repeated-measures two-way ANOVA, *P<0.05, Abbreviations: Group effect (GE).
