# Supplementary figures and images for: Unbiased analysis of spatial learning strategies in a modified Barnes maze using convolutional neural networks
Source: Sci Rep. 2024 Jul 10;14:15944. doi: 10.1038/s41598-024-66855-8 (PMC11237060; doi:10.1038/s41598-024-66855-8)

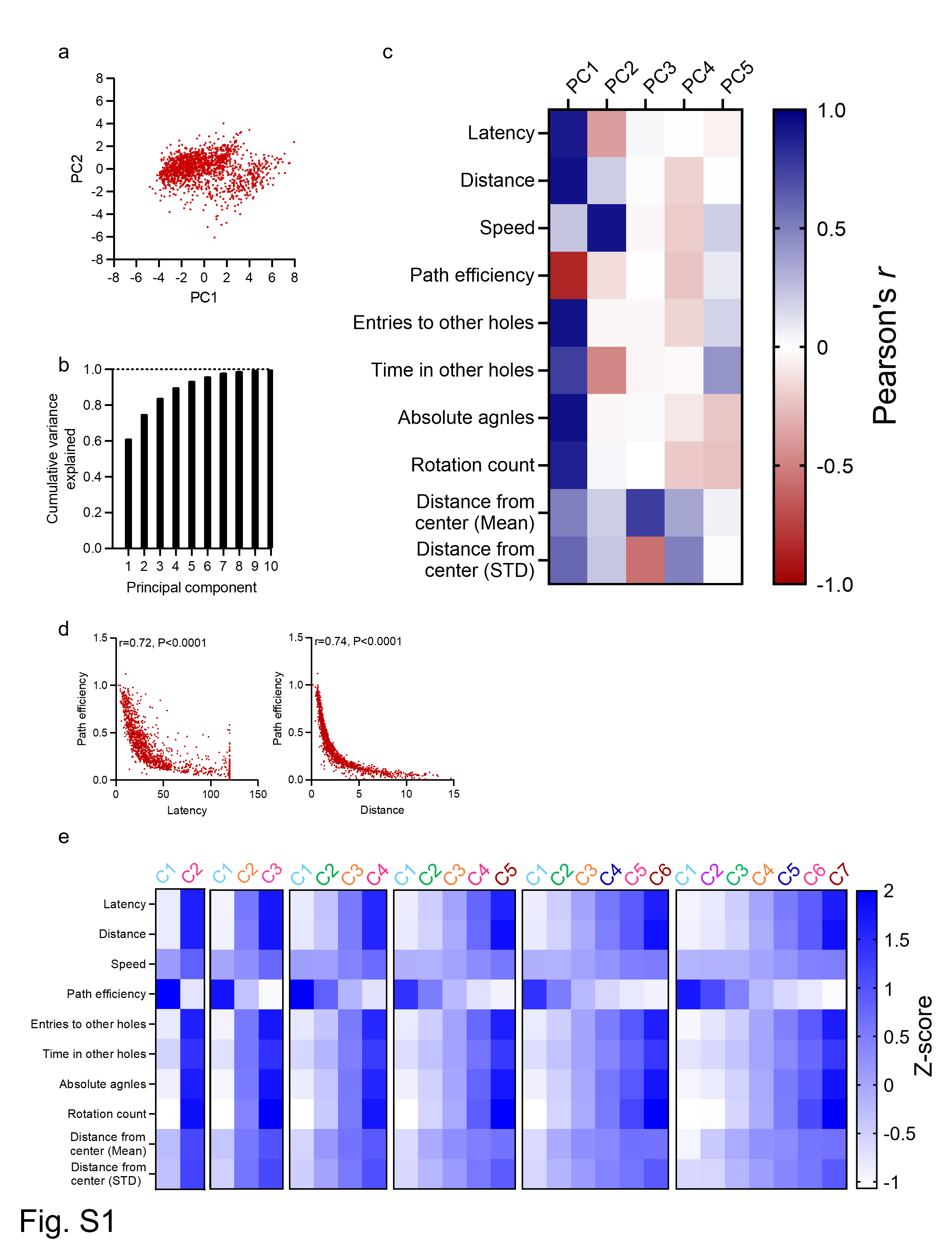

Supplement: Supplementary file 2 — Supplementary Information 2. [file 41598_2024_66855_MOESM2_ESM.tif]

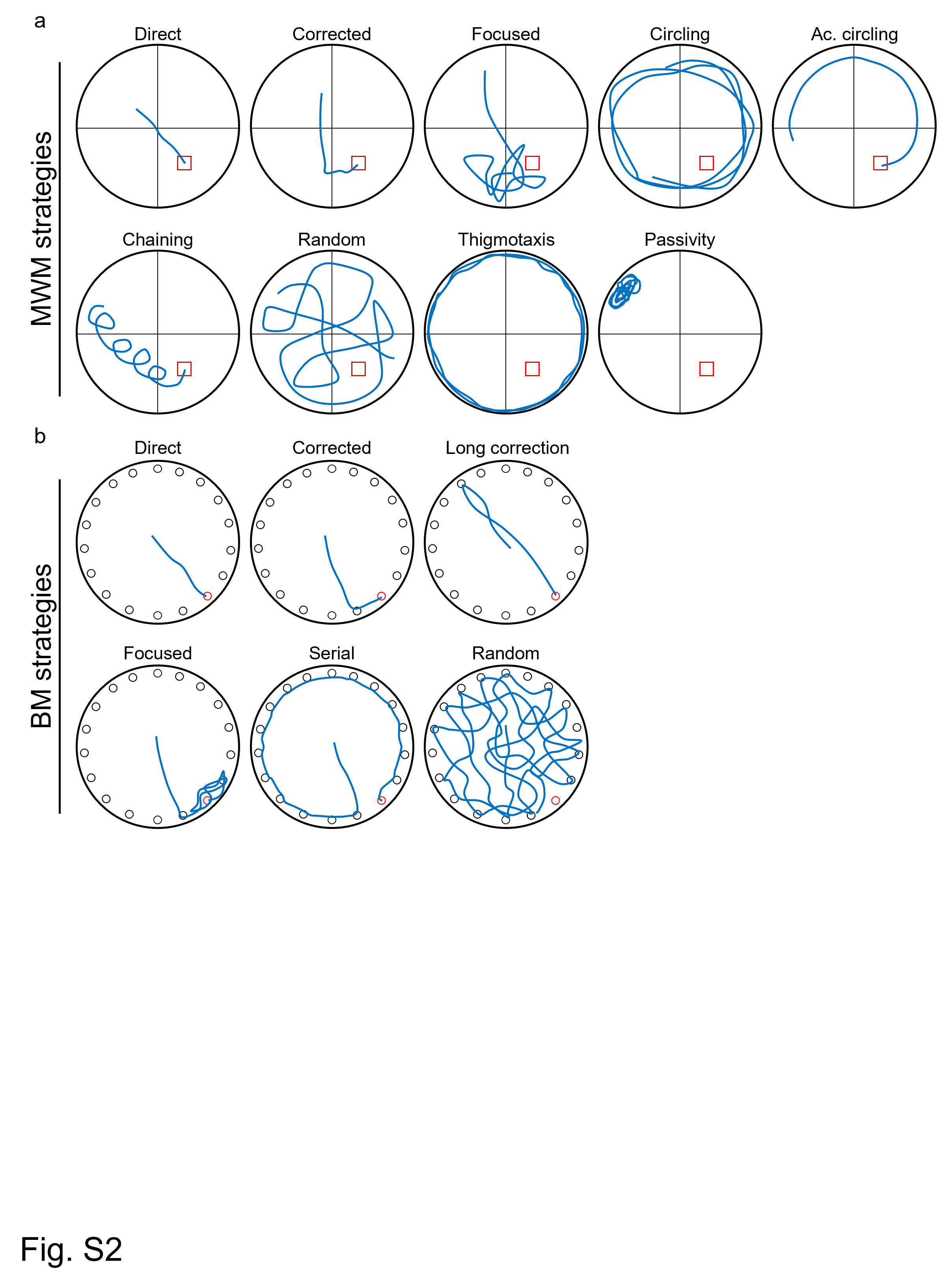

Supplement: Supplementary file 3 — Supplementary Information 3. [file 41598_2024_66855_MOESM3_ESM.tif]

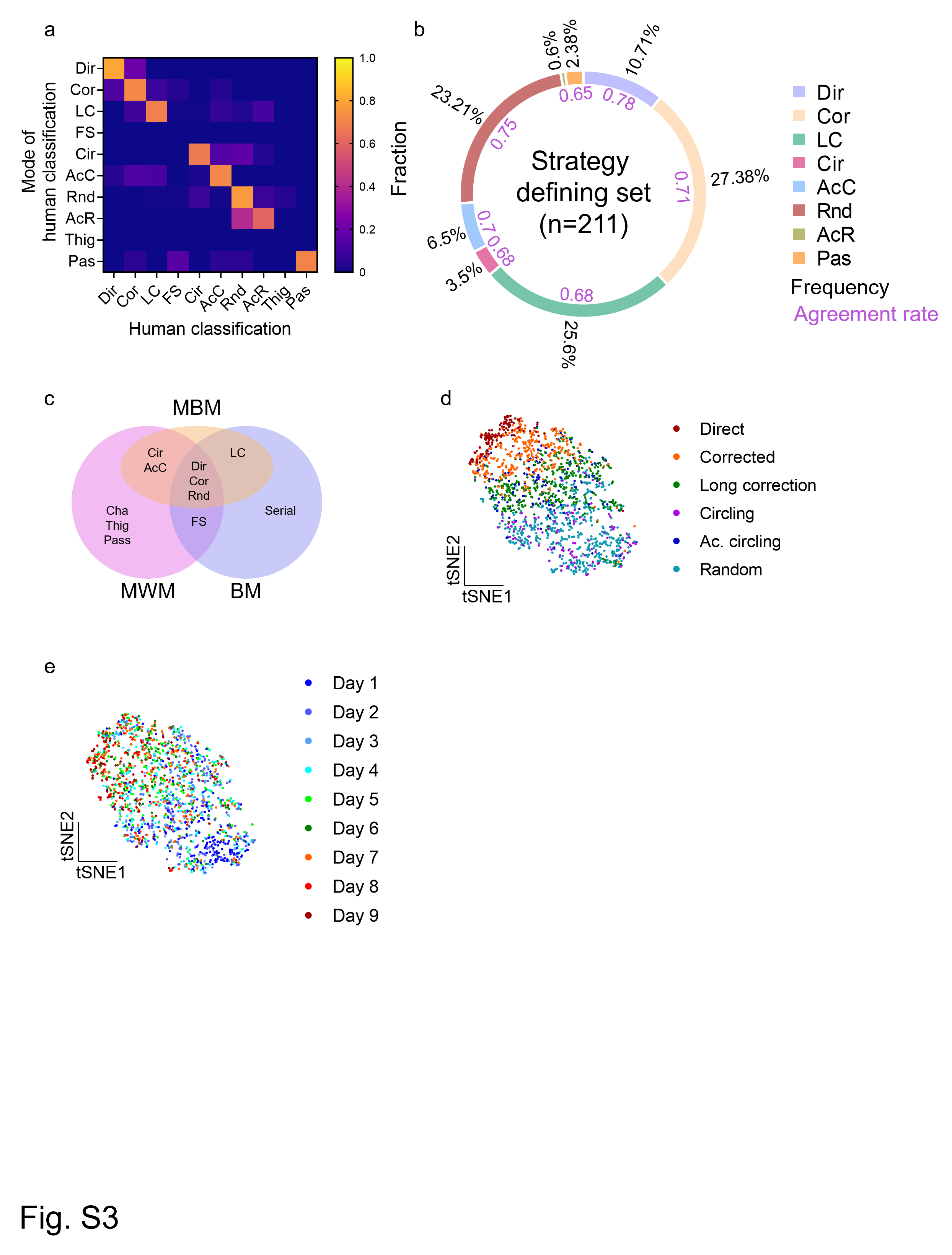

Supplement: Supplementary file 4 — Supplementary Information 4. [file 41598_2024_66855_MOESM4_ESM.tif]

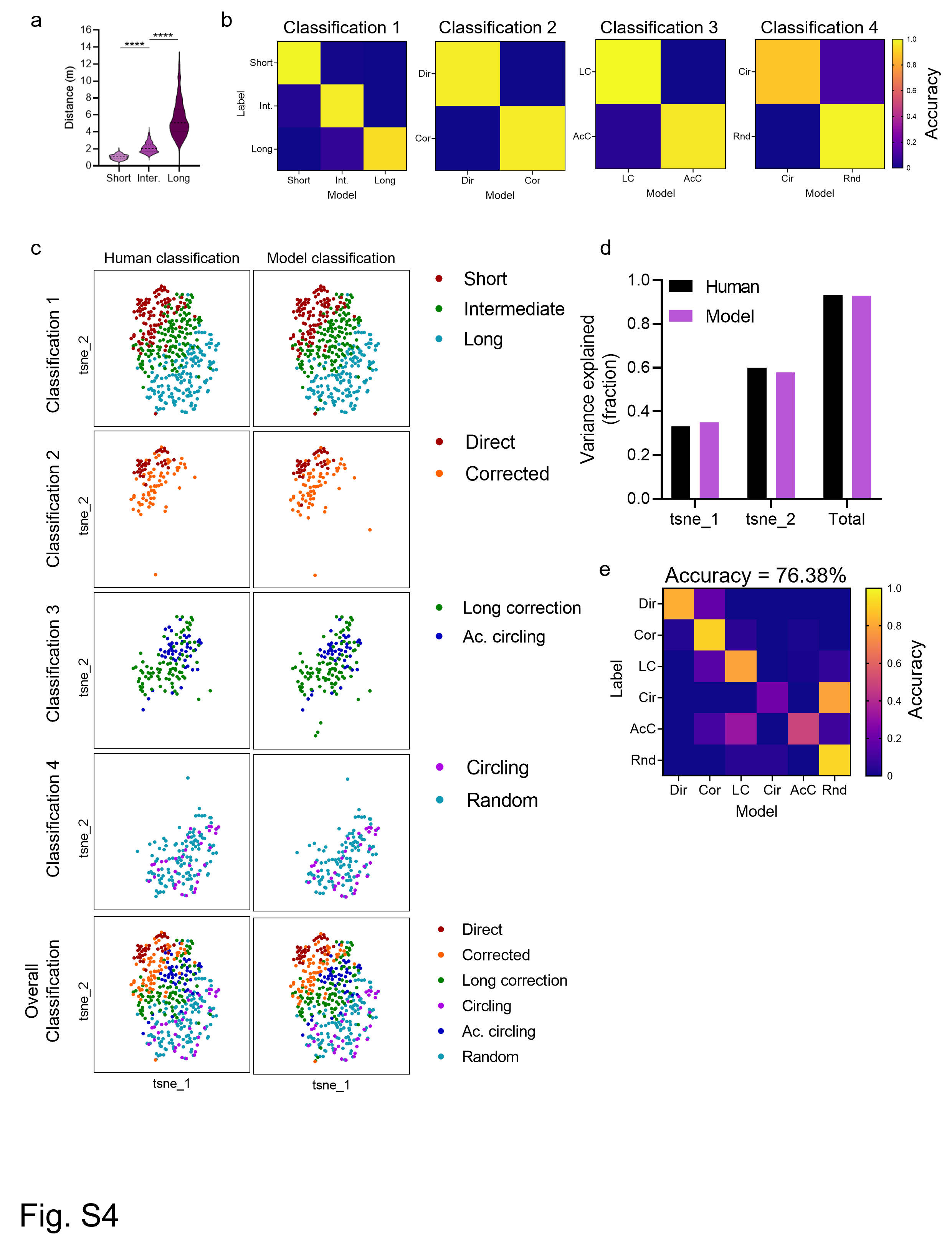

Supplement: Supplementary file 5 — Supplementary Information 5. [file 41598_2024_66855_MOESM5_ESM.tif]

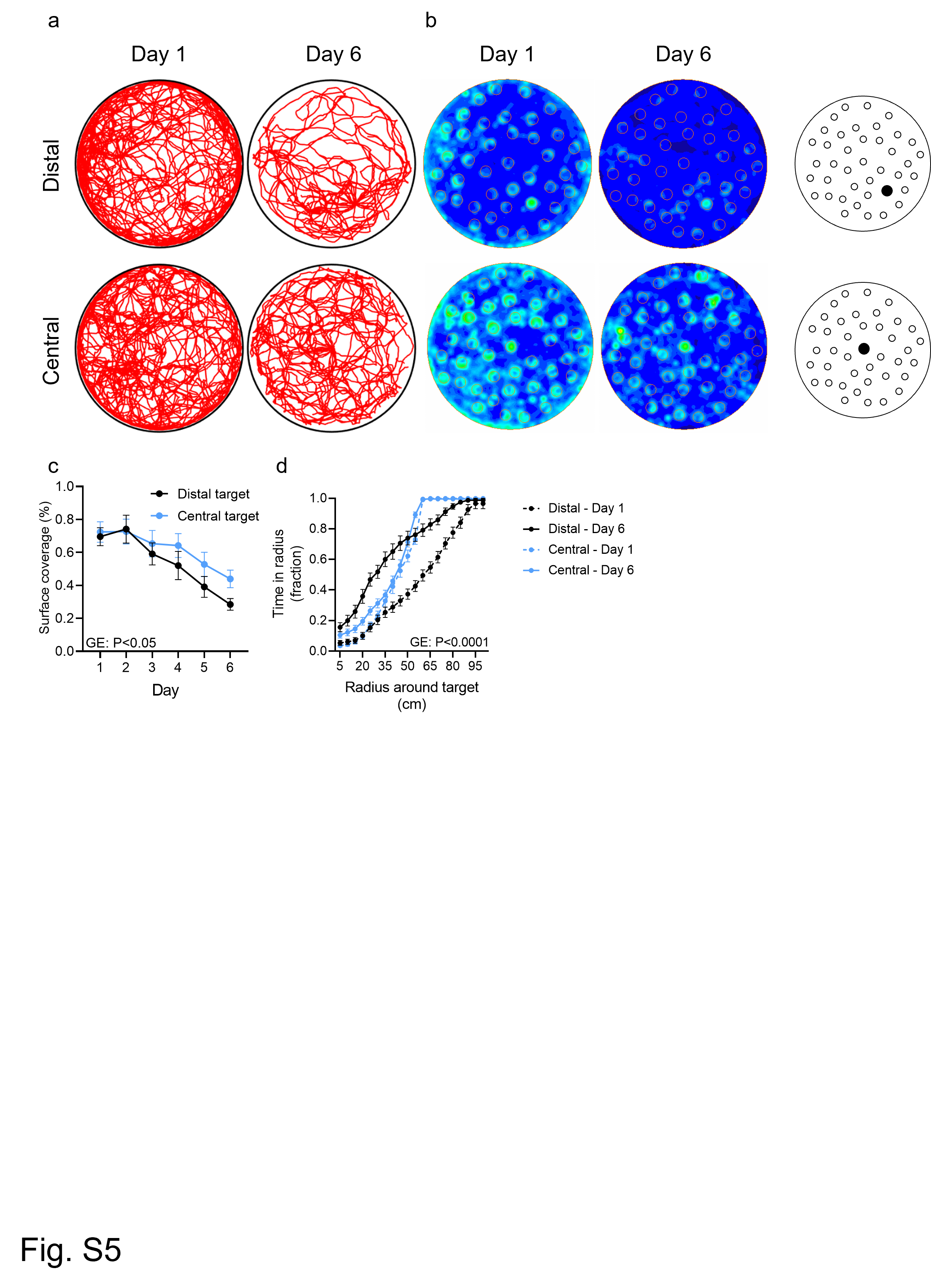

Supplement: Supplementary file 6 — Supplementary Information 6. [file 41598_2024_66855_MOESM6_ESM.tif]

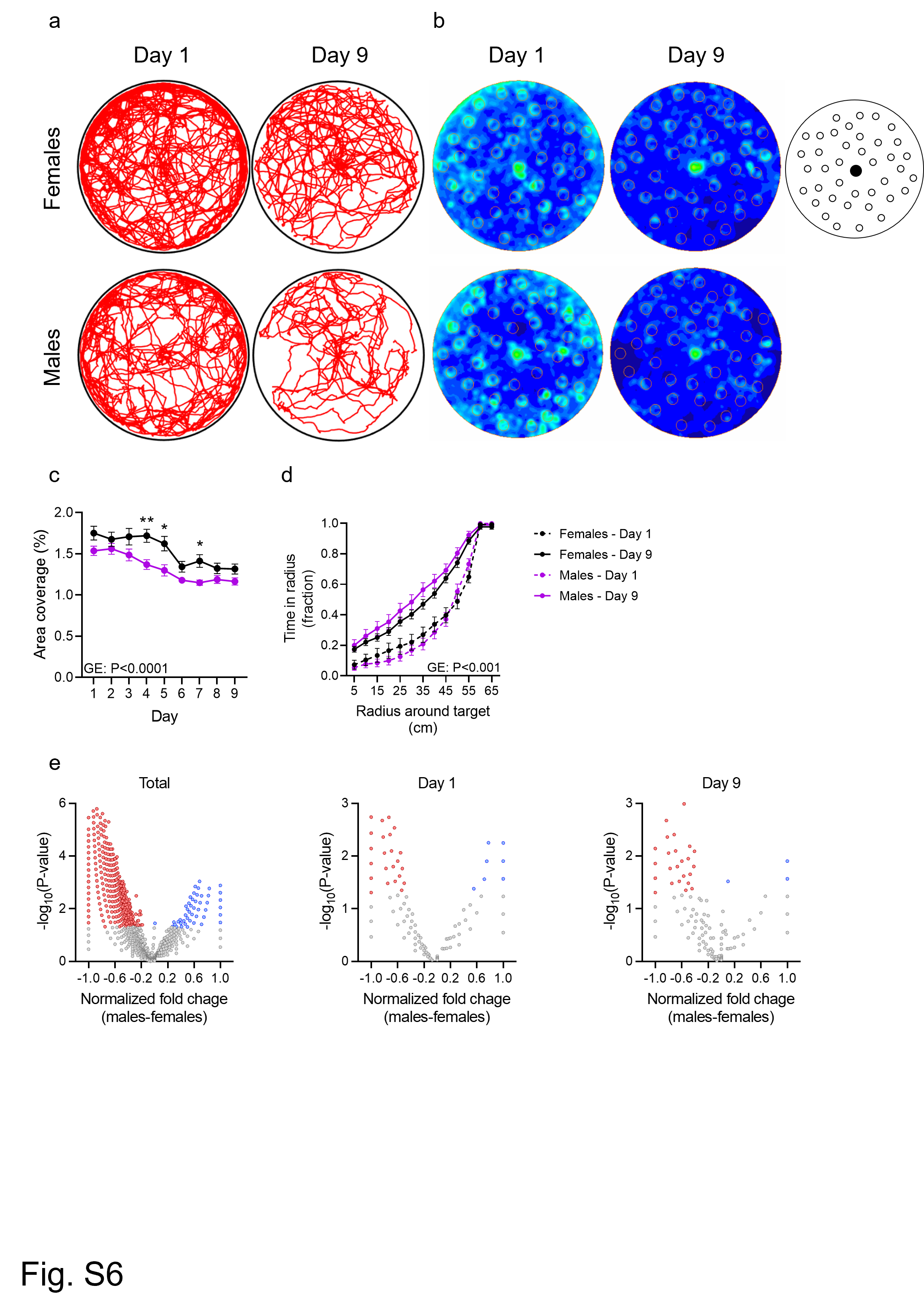

Supplement: Supplementary file 7 — Supplementary Information 7. [file 41598_2024_66855_MOESM7_ESM.tif]

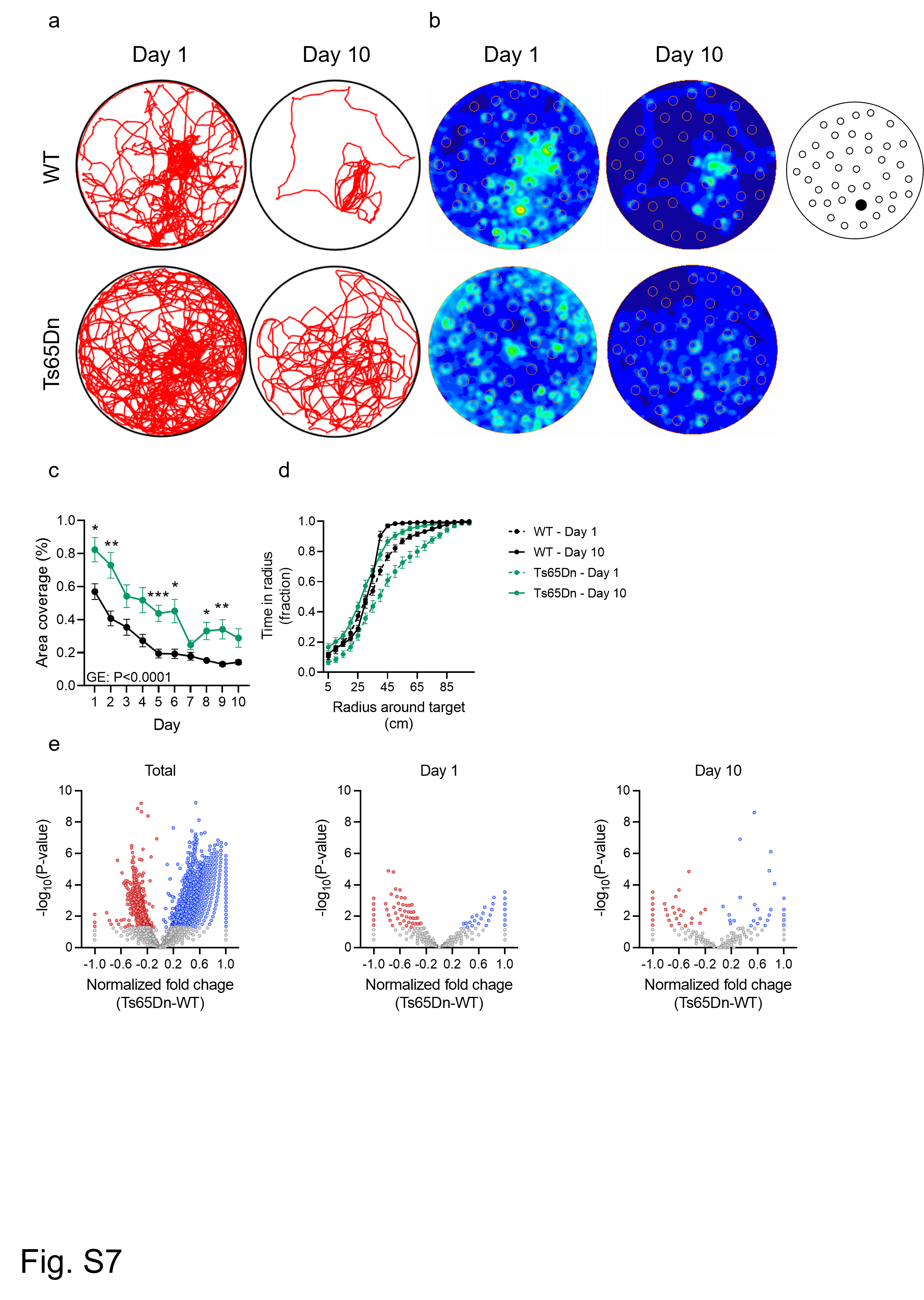

Supplement: Supplementary file 8 — Supplementary Information 8. [file 41598_2024_66855_MOESM8_ESM.tif]

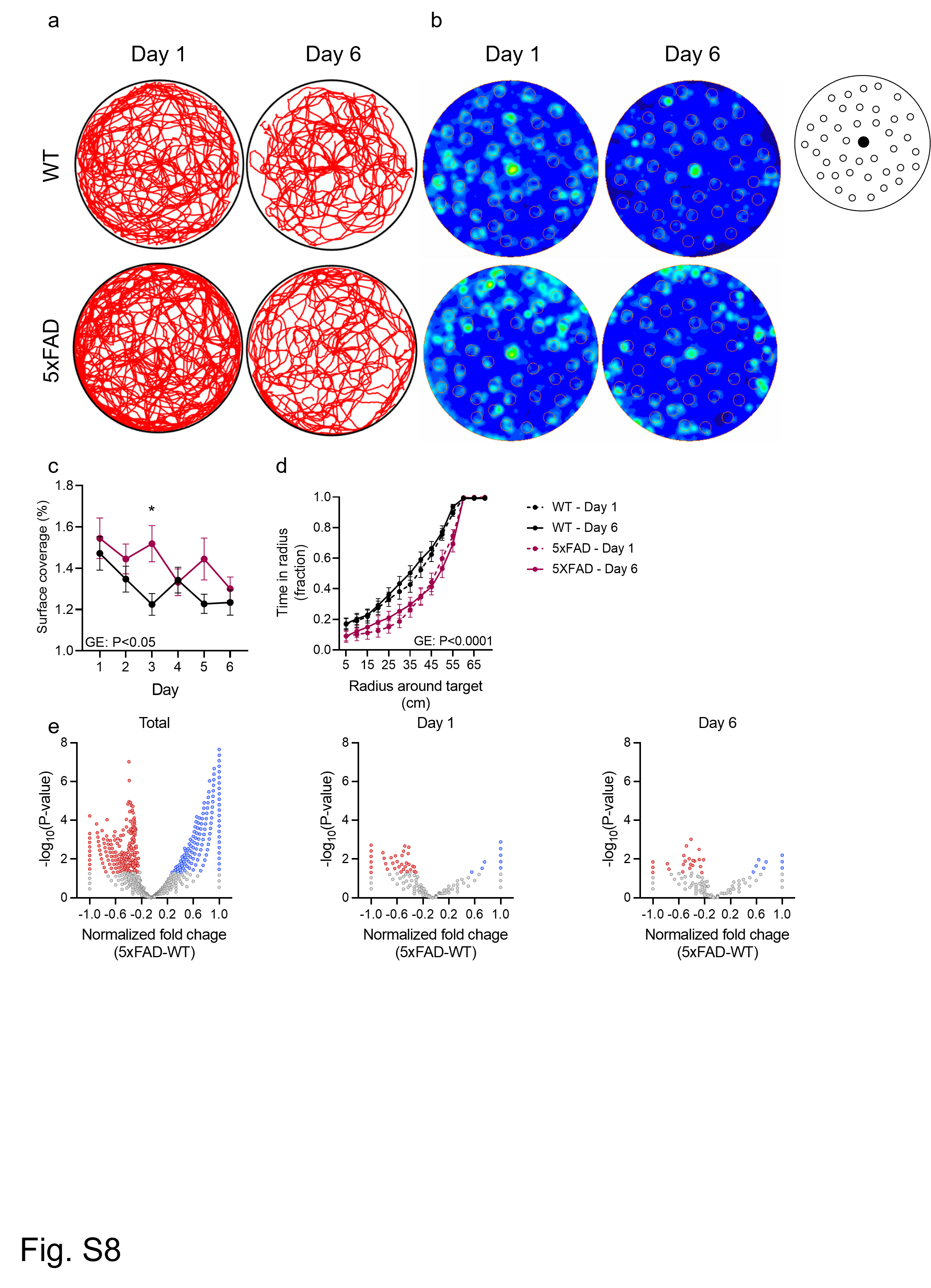

Supplement: Supplementary file 9 — Supplementary Information 9. [file 41598_2024_66855_MOESM9_ESM.tif]
